# OCT-Based Quantification and Classification of Optic Disc Structure in Glaucoma Patients

**DOI:** 10.1371/journal.pone.0160226

**Published:** 2016-08-24

**Authors:** Naoko Takada, Kazuko Omodaka, Tsutomu Kikawa, Airi Takagi, Akiko Matsumoto, Yu Yokoyama, Yukihiro Shiga, Kazuichi Maruyama, Hidetoshi Takahashi, Masahiro Akiba, Toru Nakazawa

**Affiliations:** 1 Department of Ophthalmology, Tohoku University Graduate School of Medicine, Sendai, Miyagi, Japan; 2 Topcon Corporation, Tokyo, Japan; 3 Division of Biostatistics, Tohoku University Graduate School of Medicine, Sendai, Miyagi, Japan; 4 Department of Retinal Disease Control, Ophthalmology, Tohoku University Graduate School of Medicine, Sendai, Miyagi, Japan; 5 Department of Advanced Ophthalmic Medicine, Tohoku University Graduate School of Medicine, Sendai, Miyagi, Japan; 6 Department of Ophthalmic imaging and information analytics, Tohoku University Graduate School of Medicine, Sendai, Miyagi, Japan; Bascom Palmer Eye Institute, UNITED STATES

## Abstract

**Purpose:**

To objectively classify the optic discs of open-angle glaucoma (OAG) patients into Nicolela's four disc types, i.e., focal ischemic (FI), myopic (MY), senile sclerotic (SS), and generalized enlargement (GE), with swept-source optical coherence tomography (SS-OCT).

**Methods:**

This study enrolled 113 eyes of 113 OAG patients (mean age: 62.5 ± 12.6; Humphrey field analyzer-measured mean deviation: -9.4 ± 7.3 dB). Newly developed software was used to quantify a total of 20 optic disc parameters in SS-OCT (DRI OCT-1, TOPCON) images of the optic disc. The most suitable reference plane (RP) above the plane of Bruch’s membrane opening was determined by comparing, at various RP heights, the SS-OCT-measured rim parameters and spectral-domain OCT-measured circumpapillary retinal nerve fiber layer thickness (cpRNFLT), with Pearson's correlation analysis. To obtain a discriminant formula for disc type classification, a training group of 72 eyes of 72 OAG patients and a validation group of 60 eyes of 60 OAG patients were set up.

**Results:**

Correlation with cpRNFLT differed with disc type and RP height, but overall, a height of 120 μm minimized the influence of disc type. Six parameters were most significant for disc type discrimination: disc angle (horizontal), average cup depth, cup/disc ratio, rim-decentering ratio, average rim/disc ratio (upper and lower nasal). Classifying the validation group with these parameters returned an identification rate of 80.0% and a Cohen’s Kappa of 0.73.

**Conclusion:**

Our new, objective SS-OCT-based method enabled us to classify glaucomatous optic discs with high reproducibility and accuracy.

## Introduction

Optic disc cupping and thinning of the neuronal rim are characteristic of glaucomatous optic neuropathy (GON). [1, 2] During the past decade, several objective, quantitative methods have been introduced to assess these structural changes in the optic disc and the retinal nerve fiber layer. These include red-free fundus photography, [3, 4] stereo photography, [5] scanning laser polarimetry, [6] confocal scanning laser ophthalmoscopy (Heidelberg retinal tomography: HRT), and optical coherence tomography (OCT) [2, 7–9]. Measurement parameters of optic disc cupping have shown that it is significantly associated with glaucoma severity and that, interestingly, cupping precedes visual field loss. [10, 11] Cupping is evident in high-tension glaucoma and also in normal-tension glaucoma, which is the most common type of glaucoma in Asia. In animal models, the intravitreal injection of endothelin has been found to induce retinal vessel constriction and subsequently enlarge existing cupping. [12, 13] Additionally, the cup to disc ratio (C/D ratio) has been found to be significantly associated with glaucoma severity and optic nerve head blood flow in normal-tension glaucoma (NTG). [14] Thus, the quantitative evaluation of cupping is fundamental to the investigation of glaucoma-induced structural and functional changes.

Recently, optical coherence tomography (OCT) has been undergoing rapid, significant technological improvement, and a number of OCT measurement parameters, such as circumpapillary retinal nerve fiber layer thickness (cpRNFLT) and macular layer thickness, have become helpful for glaucoma diagnosis and care. [7, 15] On the other hand, OCT measurement of cupping, including both time domain and spectral domain (SD)-OCT measurement, was for many years less helpful due to the low quality of the OCT signal in deep areas of the optic nerve head. Now, however, enhanced-depth imaging, a technique based on SD-OCT, has enabled us to observe the choroid under the retina and lamina cribrosa. [16] Another new technology, swept-source OCT (SS-OCT), uses a high-penetration laser to visualize and observe the deep structure of the ONH in detail. [17, 18] In both these new techniques, the Bruch’s membrane opening (BMO) serves as an orientation point to evaluate the structure of the optic nerve head. The BMO-minimal rim width (MRW) is a highly reproducible ONH measurement parameter with a strong potential to differentiate normal subjects from glaucoma patients. [19] New OCT technology therefore promises to improve assessment of the ONH.

The shape of the optic disc varies between individual glaucoma patients in characteristic ways, presenting disadvantages for the analysis of the overall relationship between structure and function. Nicolela et al. classified open-angle glaucoma (OAG) patients into 4 disc groups according to morphological differences. [20, 21] Nicolela found that these 4 groups also differed in clinical background and characteristics, for example, in rates of spasm, arteriosclerosis, and myopia. [20] Follow-up investigations determined clinical characteristics associated with ONH morphological type in OAG patients, including the progression of visual field loss, differences in the structure/function relationship, the susceptibility to change in cpRNFLT, and various depth in anterior surface of lamina cribrosa. [22–26] Thus, the importance of optic disc morphological characteristics lies in their relationship with clinical characteristics. However, assessment of the optic disc can sometimes be difficult because of the subjectivity of current examination techniques and variability between individuals. [21] There is thus a need for new, objective, and accurate methods of classifying the optic disc.

In this report, we describe novel software to measure optic disc parameters in SS-OCT scans of the ONH. Generally, the distance of the reference plain from the BMO is key to accurately assessing optic disc parameters. However, at present, the morphological variety of the optic disc is not considered during OCT-based quantification. Here, we describe new optic disc parameters and a new, objective method of classifying optic discs according to these parameters. We also demonstrate the accuracy of our objective classification method in an independent group of OAG patients. Our objective, highly reproducible method promises to improve not only glaucoma care, but also clinical trials of future individual treatments for glaucoma.

## Materials and Methods

### Subjects

This study included 113 eyes of 113 open angle glaucoma (OAG) patients (62.5 ± 12.6 years) with a glaucomatous visual field meeting the Anderson-Patella classification criteria. All included patients underwent testing with the Humphrey field analyzer (HFA, SITA standard 24–2) and were classifiable into 4 types according to Nicolela’s method: focal ischemic (FI), myopic (MY), senile sclerotic (SS), and generalized enlargement (GE). The distinctive characteristics of each disc type included rim notching (FI), tilted disc and temporal crescent PPA (MY), shallow cupping and halo (SS), and diffusely enlarged and round cup (GE). Four doctors specializing in glaucoma performed the classification. Patients with a normal disc size (i.e., the ratio of macular disc distance to disc diameter was 2.4–3.0) and a non-rotated disc (> 10°) were included. Patients with a spherical equivalent (SE) refractive error of < -8.00 diopters, ocular disease other than OAG, systemic disease affecting the visual field, or cataract progression were excluded. The average SE, IOP, and MD were -2.5 ± 2.6 diopters, 13.0 ± 2.6 mmHg, and -9.4 ±7.3 dB, respectively (Table 1, S1 Table).

**Table 1:** Clinical characteristics of patients by disc type.

	All	FI	MY	SS	GE	*P* value
	n = 113	n = 28	n = 30	n = 29	n = 26	
**Male / female**	49 / 64	8 / 20	15 / 15	12 / 17	14 / 12	0.238
**Age (Y)**	62.5 ± 12.6	56.3 ± 12.4	58.9 ± 12.2	70.6 ± 10.9	64.3 ± 9.9	< 0.001
**SE (D)**	-2.5 ± 2.6	-2.7 ± 2.7	-3.9 ± 2.4	-1.5 ± 1.9	-1.5 ± 2.4	0.001
**MD (dB)**	-9.4 ± 7.3	-7.8 ± 6.2	-9.0 ± 7.8	-9.6 ± 7.2	-11.3 ± 7.6	0.358
**IOP (mmHg)**	13.0 ± 2.6	12.9 ± 1.7	13.1 ± 3.5	13.0 ± 2.3	13.2 ± 2.6	0.973

A total of 113 eyes were divided into four types: FI (n = 28), MY (n = 30), SS (n = 29), and GE (n = 26). Age, SE, MD, and IOP are reported as mean ± SD. The FI group was younger than the other groups and the SS group was older (*P* < 0.001). The MY group had a lower spherical equivalent than the other groups (*P* = 0.001). There were no significant differences between the groups in MD or IOP (Kruskal-Wallis analysis). There were no significant differences between the groups in sex (Fisher’s exact test).

In order to develop a formula to classify the eyes by disc type, we enrolled 72 eyes of 72 OAG patients (62.3 ± 13.1 years, MD -8.8 ± 6.6 dB) and obtained training data (Group 1). The training data were then used to classify a validation group, comprising 60 eyes of 60 OAG patients (61.8 ± 12.9 years, MD -8.8 ± 6.8 dB) (Group 2). All eyes were also classified into Nicolela’s 4 optic disc types by 4 glaucoma specialists (Table 2, S2 Table). The inclusion and exclusion criteria were the same as described above.

**Table 2:** Clinical characteristics of 2 groups.

	Group 1	Group 2	*P* value
	n = 72	n = 60	
**Male / female**	34 / 38	31 / 29	1.000
**Age (Y)**	62.3 ± 13.1	61.8 ± 12.9	0.849
**SE (D)**	-2.3 ± 2.7	-2.4 ± 2.5	0.812
**MD (dB)**	-8.8 ± 6.6	-8.8 ± 6.8	0.995
**IOP (mmHg)**	13.5 ± 2.6	13.0 ± 2.5	0.208

Group 1, a training group including 72 eyes, was used in a stepwise regression analysis to identify parameters that could best discriminate optic disc type. Group 2, a validation group including 60 eyes, was used to validate these findings in a discriminant analysis. Age, SE, MD and IOP are reported as mean ± SD. The two groups had no significant differences in background (t test).

This study adhered to the tenets of the Declaration of Helsinki, and the protocols were approved by the Clinical Research Ethics Committee of the Tohoku University Graduate School of Medicine. (2014-1-836) Participants provided written informed consent to participate in this study. The Ethics Committee also approved this consent procedure.

### Definition of quantitative parameters of the optic nerve head

**Fig 1:**
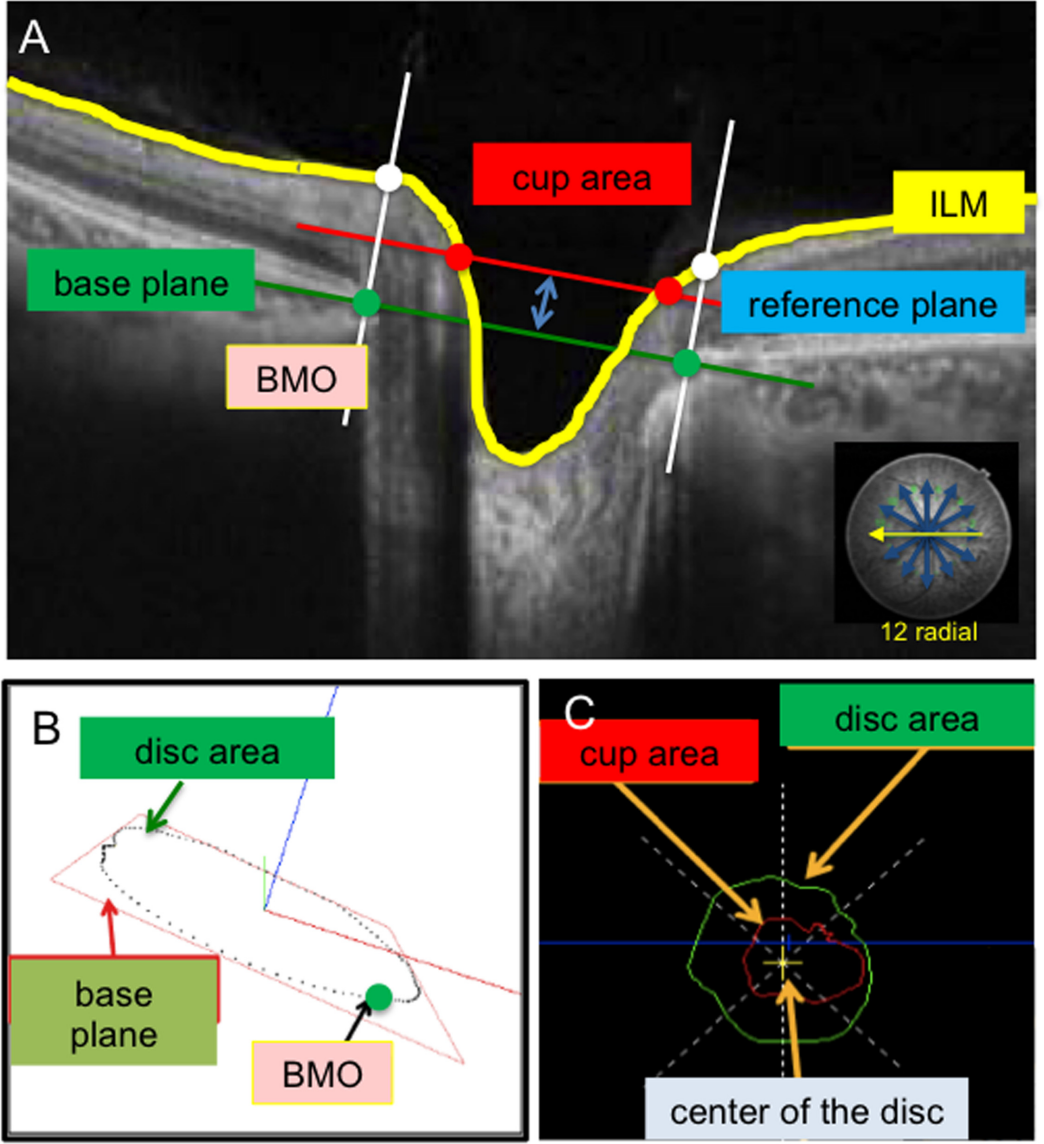
Definition of disc parameters. (A) The base plane was an approximation, drawn through the BMO points, which indicated disc area. The reference plane was set above the base plane at a height varying from 60 to180 μm (in 30 μm steps). Intersections of the ILM and the reference plane indicated the cup area. (B) Method for determining a vertical line against the base plane. (C) Definitions of cup area and disc area. ILM: inner limiting membrane. BMO: Bruch’s membrane opening.

We developed new software to analyze SS-OCT (DRI OCT-1, Topcon) images. First, 3D cube scans (resolution: 512 x 256 pixels) were obtained from a 6 x 6 mm area centered on the optic disc. The investigator then marked the BMO edges in 12 radial, reconstructed B-scan images (Fig 1A), and used the new software to draw a circular plane (Fig 1B) through the marked points, which served as the base plane. This plane was then used to define the area of the BMO (as shown in Fig 1C by the green circle). Next, the surface of the ILM (inner limiting membrane) was automatically segmented (shown in Fig 1 by the yellow line) and a reference plane was set above the base plane, varying in height from 60 to 180 μm (in 30 μm steps). The intersections of the ILM and the reference plane indicated the cup area (show in Fig 1A and 1C by the red dots and circle).

Finally, the morphological parameters of the ONH were automatically quantified in software. The disc parameters were disc area, cup area, rim area, vertical or horizontal disc diameter, vertical or horizontal C/D area ratio, maximum cup depth, average cup depth, rim to disc average in the temporal (T; 315–45 degrees), superotemporal (TS; 45–90 degrees), superonasal (NS; 90–135 degrees), nasal (N; 135–225 degrees), inferonasal (NI; 225–270 degrees), and inferotemporal (TI; 270–315 degrees) sectors (these sectors are also used in HRT), rim decentering ratio, horizontal disc angle, and disc height difference.

The rim decentering ratio was calculated with the following formula: (rim area in sector TS-TI)/(rim area in sector TS+TI). This parameter indicates decentering of the cup. The horizontal disc angle was defined as the angle between a horizontal line and the line through the temporal and nasal BMO points. Disc height difference was the difference in the length of a vertical line reaching the ILM through the BMO points between the temporal and nasal areas.

### Statistical analysis

In order to determine the most suitable reference plane to examine the cup area, Pearson's correlation analysis was used to determine the correlation between SS-OCT-measured rim parameters and SD-OCT-measured cpRNFLT at various reference plane heights between 60 and 180 μm. A linear regression analysis was then used to determine factors affecting optic disc type in the rim area. We identified the intra-rater and the inter-rater reliability of this method by calculating the intra-class correlation coefficient (ICC).

A stepwise regression analysis was used to determine the parameters best able to identify optic disc type in the 72-eye training group, with the results then being tested in the 60-eye validation group, in a discriminant analysis. In this discriminant analysis the probability of belonging to each disc type was calculated, and the highest score decided the disc type. A receiver operating characteristic (ROC) analysis was performed to determine whether the calculated probability of belonging to a particular disc type had successfully identified the type, using the disc type classification by the specialists as a reference.

## Results

### Setting the most suitable reference plane

The eyes were divided into four groups according to disc type: FI (28 eyes), MY (30 eyes), SS (29 eyes), and GE (26 eyes). The FI group was younger than the other groups, and the SS group was older (both *P* < 0.001). The MY group had a lower refractive error than the other groups (*P* = 0.001). There were no significant differences between the groups in MD or IOP (*P* > 0.05, Table 1).

**Fig 2:**
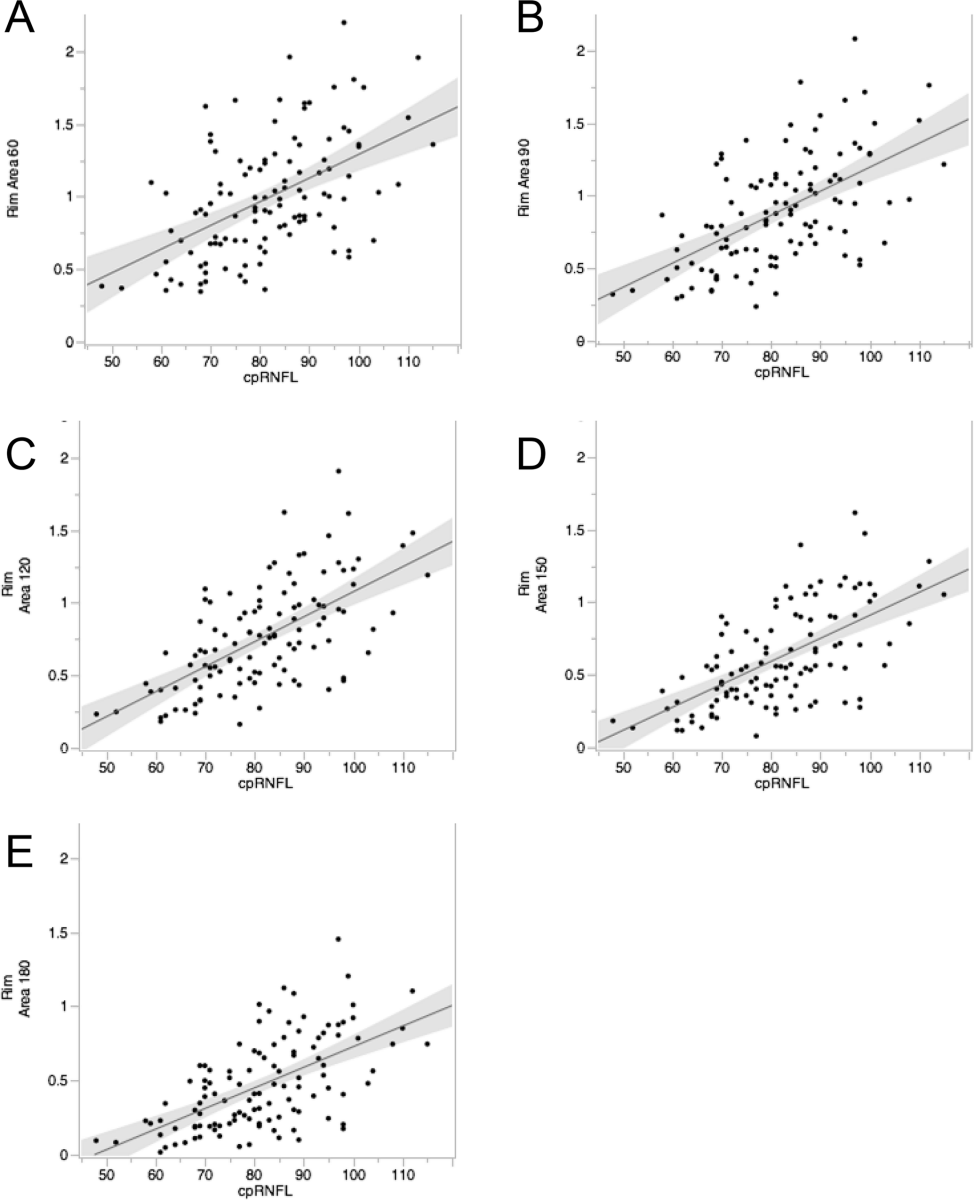
The relationship between rim area and cpRNFLT when the reference plane was set at different heights. (Pearson’s product/moment correlation coefficient) Scatter plot showing the relationship between rim area and cpRNFLT at reference plane height of 60 (A), 90 (B), 120 (C), 150 (D), and 180 (E) μm. The highest correlation between rim area and cpRNFLT was at 120 μm in the overall group of subjects.

Fig 2 shows the relationship between rim area and cpRNFLT when the reference plane was set at different heights. The correlation coefficient was 0.525 at a height of 60 μm (*P* < 0.001), 0.575 at 90 μm (*P* < 0.001), 0.625 at 120 μm (*P* < 0.001), 0.622 at 150 μm (*P* < 0.001), and 0.595 at 180 μm (*P* < 0.001). The reference plane height with the highest correlation between rim area and cpRNFLT was thus 120 μm in the overall group of subjects (Fig 2C).

**Fig 3:**
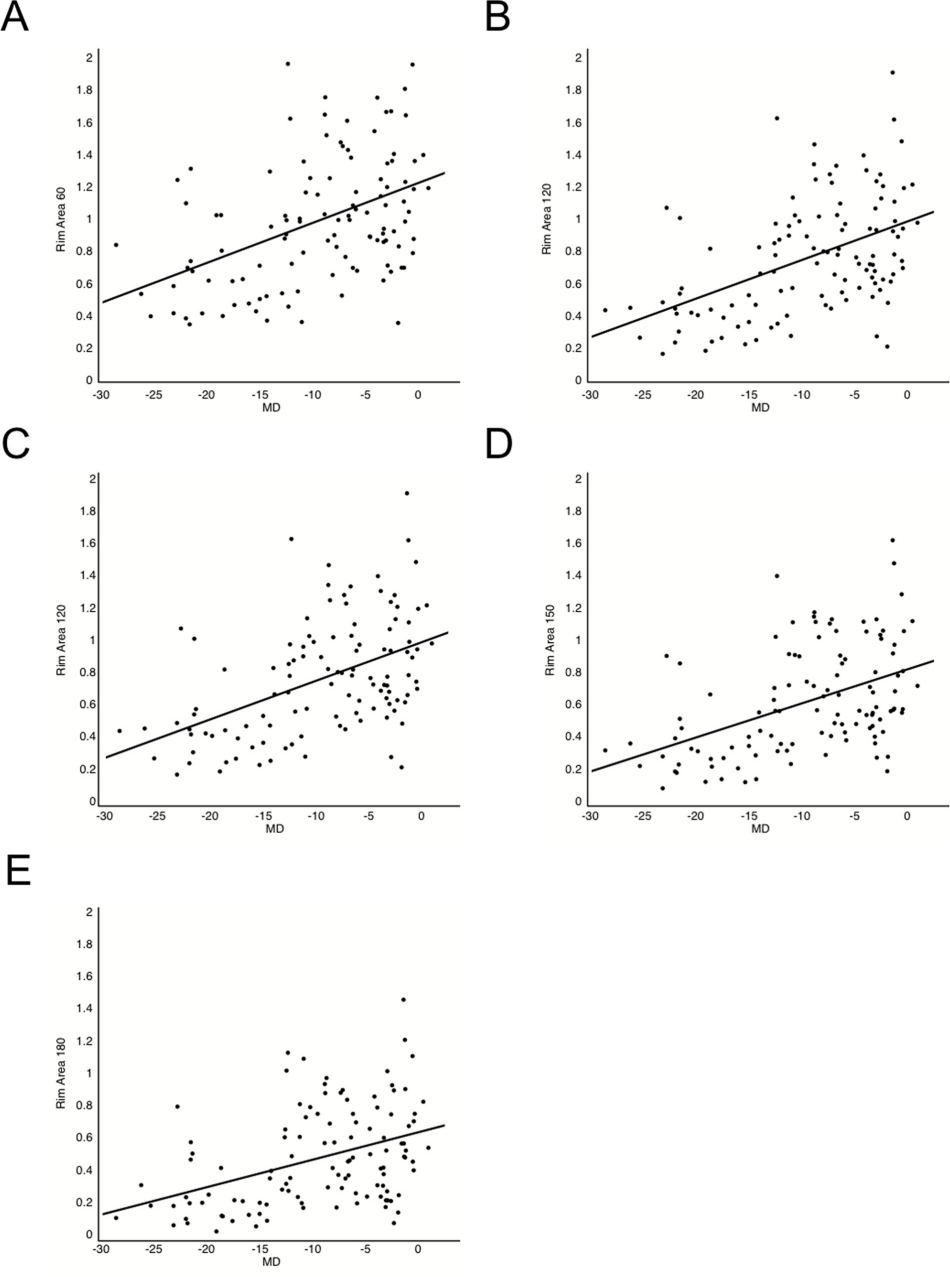
The relationship between HFA MD and rim area when the reference plane was set at different heights. (Pearson’s product/moment correlation coefficient) Scatter plot showing the relationship between rim area and HFA MD at reference plane height of 60 (A), 90 (B), 120 (C), 150 (D), and 180 (E) μm. The highest correlation between rim area and HFA MD was at 120 μm in the overall group of subjects.

Fig 3 shows the relationship between HFA MD and rim area when the reference plane was set at different heights. The correlation coefficient was 0.441 at a height of 60 μm (*P* < 0.001), 0.472 at 90 μm (*P* < 0.001), 0.481 at 120 μm (*P* < 0.001), 0.460 at 150 μm (*P* < 0.001), and 0.404 at 180 μm (*P* = 0.001). The reference plane height with the highest correlation between rim area and HFA MD was thus 120 μm in the overall group of subjects (Fig 3C).

Each disc type had a different correlation coefficient. Generally, the GE group had a higher correlation coefficient than the other groups (Table 3). The reference plane height with the highest correlation between rim area and cpRNFLT was 120 μm in the FI group (r = 0.511, *P* = 0.006), 150 μm in the MY group (r = 0.575, *P* = 0.007), 180 μm in the SS group (r = 0.551, *P* = 0.017), and 60 μm in the GE group (r = 0.805, *P* < 0.001) (Table 3).

**Table 3:** The correlation coefficient between rim area and cpRNFLT.

Reference plane height	All	FI	MY	SS	GE
**60 μm**	0.525	0.425	0.400	0.439	0.805
	(*P* < 0.001)	(*P* = 0.024)	(*P* = 0.001)	(*P* = 0.002)	(*P* < 0.001)
**90 μm**	0.575	0.453	0.483	0.513	0.793
	(*P* < 0.001)	(*P* = 0.016)	(*P* = 0.001)	(*P* = 0.002)	(*P* < 0.001)
**120 μm**	0.625	0.511	0.566	0.547	0.776
	(*P* < 0.001)	(*P* = 0.006)	(*P* = 0.001)	(*P* = 0.002)	(*P* < 0.001)
**150 μm**	0.622	0.505	0.575	0.549	0.780
	(*P* < 0.001)	(*P* = 0.006)	(*P* = 0.007)	(*P* = 0.004)	(*P* < 0.001)
**180 μm**	0.595	0.470	0.572	0.551	0.707
	(*P* < 0.001)	(*P* = 0.012)	(*P* = 0.028)	(*P* = 0.017)	(*P* < 0.001)

The correlation coefficient between rim area and cpRNFLT at different reference plane heights and with different disc types. (Pearson’s product/moment correlation coefficient) The highest correlations were found at a height of 120 μm in the FI group (*r* = 0.511, *P* = 0.006), 150 μm in the MY group (*r* = 0.575, *P* = 0.007), 180 μm in the SS group (*r* = 0.551, *P* = 0.017), and 60 μm in the GE group (*r* = 0.805, *P* < 0.001).

Thus, optic disc type influenced the most appropriate height of the reference plane. Next, we investigated the most suitable reference height independent of disc type. We found that the rim area at a reference plane height of 120 μm was significantly influenced by cpRNFLT, but not affected by disc type, in a linear regression analysis (F value = 1.96, *P* = 0.1236, Table 4). A height of 120 μm was the most independent from disc type. Thus, we found that 120 μm was the most suitable height for OCT evaluation of cupping parameters.

**Table 4:** Linear regression analysis of cpRNFLT and disc type influence on rim area.

Reference plane height		F value	*P* value	R^2^	CV
**60 μm**	cpRNFLT	39.08	< 0.001	0.33	34.32
	Disc type	3.06	0.031		
**90 μm**	cpRNFLT	49.84	< 0.001	0.38	33.95
	Disc type	2.92	0.037		
**120 μm**	cpRNFLT	62.54	< 0.001	0.42	36.87
	Disc type	1.96	0.124		
**150 μm**	cpRNFLT	64.31	< 0.001	0.44	41.41
	Disc type	3.05	0.032		
**180 μm**	cpRNFLT	55.26	< 0.001	0.42	50.51
	Disc type	3.88	0.011		

In all disc types, rim area was significantly influenced by cpRNFLT at a reference plane height of 120 μm, but not by disc type (F value = 1.96, *P* = 0.124).

### The reproducibility of measuring disc parameters

Our new software output a total of 20 disc parameters based on SS-OCT measurements. Two testers marked the BMO points and ILM in images from 20 patients selected at random. The disc parameters were then calculated and their reproducibility was determined, including both intra-rater and inter-rater reliability. This analysis showed that SS-OCT parameters quantified by our method had high reproducibility (Table 5, S3 Table).

**Table 5:** List of SS-OCT measurement parameters and their reproducibility.

Parameter (20 patients)	Inter-rater ICC	Intra-rater ICC
**Disc Area**	0.95	0.98
**Cup Area**	0.99	0.99
**Rim Area**	0.92	0.97
**Disc Dia (V)**	0.91	0.94
**Disc Dia (H)**	0.91	0.94
**C/D Ratio (V)**	0.91	0.95
**C/D Ratio (H)**	0.91	0.96
**C/D Ratio (Area)**	0.95	0.99
**R/D Ratio (Area)**	0.95	0.99
**Max Cup Depth**	0.99	0.99
**Avg Cup Depth**	0.99	0.99
**R/D Avg (SecT)**	0.93	0.96
**R/D Avg (SecTS)**	0.91	0.97
**R/D Avg (SecNS)**	0.95	0.95
**R/D Avg (SecN)**	0.94	0.96
**R/D Avg (SecNI)**	0.89	0.93
**R/D Avg (SecTI)**	0.92	0.95
**Rim Decentering Ratio**	0.86	0.91
**Disc Angle (H)**	0.98	0.98
**Disc Height Difference**	0.78	0.92

Two testers marked the BMO points and ILM in 20 patients selected at random. Morphological parameters were quantified and their reproducibility was assessed, both for intra-rater (i.e., between the two testers) and inter-rater (i.e., between two ratings of the same tester) reliability. All parameters had an ICC > 0.7.

The measurement parameters obtained from SS-OCT images of patients with different disc types with our new software reflected the characteristics previously described by Nicolela, such as a small cup area in FI eyes, a high disc angle in MY eyes, a shallow cup in SS eyes, and a large, deep cup in GE eyes (Table 6).

**Table 6:** Measurement parameters obtained with new SS-OCT software.

Parameter	All	FI	MY	SS	GE	*P* value
Disc Area	2.48 ± 0.62	2.36 ± 0.41	2.51 ± 0.79	2.52 ± 0.59	2.52 ± 0.63	0.826
Cup Area	1.72 ± 0.62	1.49 ± 0.45	1.66 ± 0.67	1.82 ± 0.61	1.91 ± 0.68	0.027
Rim Area	0.76 ± 0.36	0.87 ± 0.32	0.85 ± 0.35	0.70 ± 0.37	0.60 ± 0.36	0.007
Disc Dia (V)	0.92 ± 0.11	0.90 ± 0.07	0.93 ± 0.14	0.92 ± 0.12	0.91 ± 0.11	0.997
Disc Dia (H)	0.86 ± 0.12	0.84 ± 0.09	0.86 ± 0.15	0.87 ± 0.10	0.88 ± 0.12	0.608
C/D Ratio (V)	0.87 ± 0.12	0.87 ± 0.10	0.85 ± 0.13	0.87 ± 0.14	0.88 ± 0.12	0.517
C/D Ratio (H)	0.89 ± 0.10	0.85 ± 0.09	0.88 ± 0.11	0.92 ± 0.09	0.92 ± 0.12	0.003
C/D Ratio (Area)	0.69 ± 0.15	0.63 ± 0.13	0.66 ± 0.12	0.71 ± 0.14	0.75 ± 0.16	0.001
R/D Ratio (Area)	0.31 ± 0.15	0.37 ± 0.13	0.34 ± 0.11	0.29 ± 0.14	0.25 ± 0.16	0.001
Max Cup Depth	567 ± 173	562 ± 135	563 ± 174	479 ± 127	674 ± 199	< 0.001
Avg Cup Depth	330 ± 137	330 ± 128	309 ± 122	257 ± 97	437 ± 140	< 0.001
R/D Avg (SecT)	0.08 ± 0.06	0.11 ± 0.07	0.08 ± 0.05	0.06 ± 0.06	0.06 ± 0.06	0.001
R/D Avg (SecTS)	0.11 ± 0.06	0.14 ± 0.05	0.13 ± 0.07	0.09 ± 0.05	0.08 ± 0.06	< 0.001
R/D Avg (SecNS)	0.11 ± 0.06	0.12 ± 0.06	0.13 ± 0.06	0.09 ± 0.05	0.09 ± 0.07	0.080
R/D Avg (SecN)	0.10 ± 0.07	0.11 ± 0.06	0.11 ± 0.06	0.10 ± 0.07	0.07 ± 0.08	0.029
R/D Avg (SecNI)	0.08 ± 0.06	0.08 ± 0.05	0.09 ± 0.06	0.09 ± 0.08	0.06 ± 0.07	0.127
R/D Avg (SecTI)	0.07 ± 0.06	0.08 ± 0.06	0.08 ± 0.07	0.07 ± 0.08	0.06 ± 0.06	0.185
Rim Decentering Ratio	0.29 ± 0.40	0.32 ± 0.27	0.29 ± 0.32	0.31 ± 0.53	0.22 ± 0.42	0.732
Disc Angle (H)	4.07 ± 3.60	3.30 ± 2.78	6.99 ± 3.76	2.95 ± 2.51	2.77 ± 3.50	< 0.001
Disc Height Difference	240 ± 68	260 ± 59	251 ± 87	224 ± 65	224 ± 51	0.103

SS-OCT based morphological parameters are reported as mean ± SD. They reflected the characteristics of each disc type described by Nicolela, including a small cup area and thinning in the inferior rim area in the FI disc type group, a higher disc angle in the MY group, a shallow cup and thinning in all areas in the SS group, and a large, deep cup in the GE group. (Kruskal-Wallis analysis)

### Establishment of a formula to classify the different disc types and validation of the formula

We measured optic disc parameters in a training group of 72 eyes (Group 1) in order to obtain data for the development of a formula to differentiate disc type. We then tested this formula in a validation group of 60 eyes (Group 2). Four glaucoma specialists then classified the eyes in both groups into Nicolela’s 4 disc types by unanimous agreement. Clinical background, including sex, age, SE, HFA MD, and IOP did not significantly differ between the groups (Table 7).

**Table 7:** Clinical background for the training group (Group 1) and validation group (Group 2), classified by 4 glaucoma specialists.

	All	FI	MY	SS	GE	*P* value
Group 1	n = 72	n = 18	n = 18	n = 18	n = 18	
Age (Y)	62.3 ± 13.1	64.4 ± 12.8	57.7 ± 12.1	67.6 ± 13.1	59.7 ± 13.3	0.047
SE (D)	-2.3 ± 2.7	-1.4 ± 2.6	-4.5 ± 1.9	-2.1 ± 2.4	-1.3 ± 2.4	< 0.001
MD (dB)	-8.8 ± 6.6	-5.9 ± 5.0	-8.1 ± 5.5	-10.4 ± 8.1	-10.9 ± 6.6	0.104
IOP (mmHg)	13.5 ± 2.6	13.0 ± 2.1	13.7 ± 2.8	13.6 ± 2.3	13.8 ± 3.3	0.694
Group 2	n = 60	n = 15	n = 15	n = 15	n = 15	
Age (Y)	62.6 ± 12.3	54.6 ± 14.2	57.0 ± 13.4	74.7 ± 5.3	61.3 ± 6.0	< 0.001
SE (D)	-2.4 ± 2.5	-2.9 ± 2.0	-4.3 ± 2.5	-0.9 ± 2.2	-1.7 ± 1.9	< 0.001
MD (dB)	-9.2 ± 7.2	-6.2 ± 3.8	-9.1 ± 8.4	-9.2 ± 6.0	-10.8 ± 8.0	0.501
IOP (mmHg)	13.0 ± 2.4	12.7 ± 1.3	13.3 ± 3.5	12.5 ± 2.1	13.4 ± 2.7	0.880

(Group 1) There were significant differences in age (*P* = 0.047) and SE (*P* < 0.001).

(Group 2) There were significant differences in age (*P* < 0.001) and SE (*P* < 0.001).

(*P*-values were calculated using the Kruskal-Wallis analysis)

**Fig 4:**
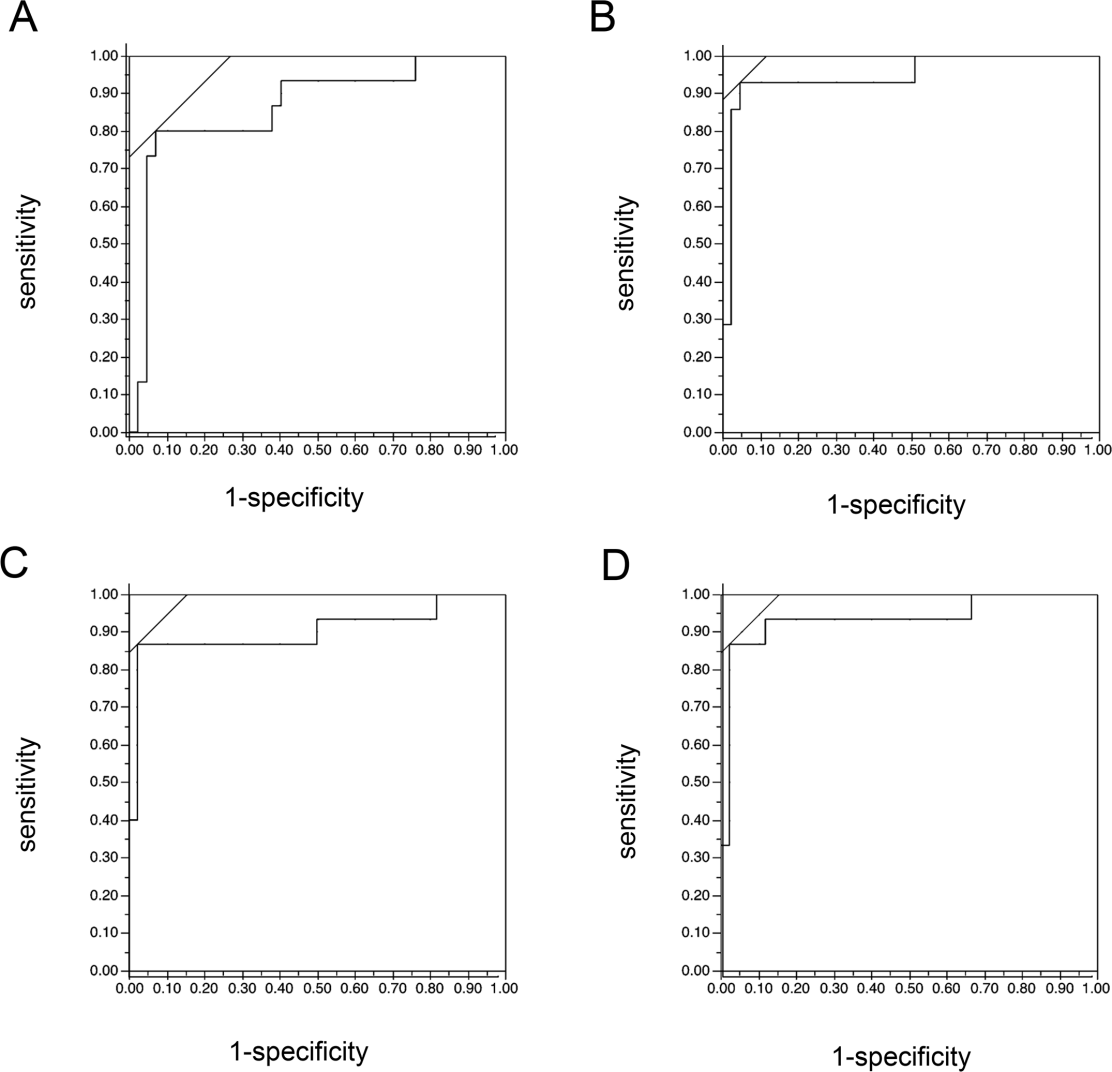
The ROC for successful identification of disc type. A ROC analysis was performed to determine whether the disc type had been successfully identified. A. FI: 0.84, B. GE: 0.93, C. MY: 0.89, D. SS: 0.93.

A stepwise analysis revealed that the following 6 parameters were most useful for classifying eyes in the training group: disc angle (horizontal) (F power = 17.92, *P* < 0.001), average cup depth (F power = 24.00, *P* < 0.001), R/D average (NS, NI) (F power = 1.77, 6.50, *P* = 0.16, < 0.001, respectively), R/D ratio (area) (F power = 8.51, *P* < 0.001), and rim decentering ratio (F power = 2.57, *P* = 0.062). In this discriminant analysis, the probability of belonging to each disc type was calculated, and the disc type was then decided according to the highest score. The identification rate with the classification performed by the glaucoma specialists was 81.94%. Cohen’s Kappa was 0.76. Next, the eyes in Group 2, the validation group, were classified using the parameters listed above, and the classification was evaluated with a discriminant analysis. This analysis calculated the most probable disc type. The ratio of formula-predicted disc types to specialist-detected disc types were FI: 12/15, GE: 12/15, MY: 11/15, and SS: 13/15. The identification rates with the classifications performed by the specialists were thus, 80.0% for FI, 80.0% for GE, 73.3% for MY, 87.7% for SS and 80.0% for overall. The validation group included 24 early-stage OAG patients (-6.0 ≤ MD), 19 middle-stage OAG patients (-12.0 ≤ MD < -6.0), and 17 late-stage OAG patients (MD < -12.0). We also analyzed a subgroup containing only the early-stage patients, and found that the ratios of formula-predicted disc types to specialist-detected disc type were FI: 5/7, GE: 4/5, MY: 5/7, SS: 4/5, with a total identification rate of 81.8%. A ROC analysis was performed to determine whether the calculated probability of belonging to a particular disc type matched the correct disc type, based on a comparison with classification performed by specialists. The ROC for successful identification of disc type is shown in Fig 4. This statistical classification had a high area under the ROC (AUC) in each disc type: 0.84 for FI, 0.93 for GE, 0.89 for MY, and 0.93 for SS. The sensitivity was 0.80 for FI, 0.87 for GE, 0.87 for MY, and 0.87 for SS. The specificity was 0.91 for FI, 0.96 for GE, 0.96 for MY, and 0.96 for SS. Cohen’s Kappa was 0.73. (Fig 4)

## Discussion

In this study, we developed new software to quantify optic disc topography in SS-OCT images. We found that setting a reference plane set 120 μm above the BMO minimized the influence of disc type and allowed us to obtain the most accurate measurements. We found that each of the 20 ONH topographical parameters output by our software had excellent reproducibility. We used these parameters to classify a group of glaucomatous eyes by optic disc type, and found that this classification correlated well with classifications based on a previous, HRT II-based method [11]. A stepwise analysis showed that the following 6 parameters were most useful for classifying the ONH: disc angle (horizontal), average cup depth, average rim to disc ratio (NS, NI), rim decentering ratio, and R/D ratio. Finally, a discriminant analysis based on separate training and validation groups showed that objective classification of the ONH with our method closely matched subjective classification performed by glaucoma specialists.

In existing techniques for the examination of retinal morphology, the most suitable height for the reference plane depends on the equipment used. Heidelberg retinal tomography uses a standard of 50 μm posterior to the mean contour line. [27] Stratus OCT (Carl Zeiss) uses a height of 150 μm above the RPE to obtain the largest AUC, the highest correlation with visual function, and the most accurate detection of early glaucoma. [28] In this study, we confirmed that cupping depth differed significantly between patients with the SS disc type (i.e., a shallower disc) and the GE disc type (i.e., a deeper disc). This finding was reasonable, because structural changes in the ONH, especially cupping depth, have long been used for disc classification. Importantly, this study revealed that the reference plane height with the highest correlation coefficient between rim area and cpRNFLT differed with disc type. Thus, in order to obtain the most accurate topographical data for the disc, it is critical to minimize the influence of disc type by choosing the appropriate reference plane height. Research into the relationship between the structure and function of the eye in glaucoma may benefit from this insight into the impact of disc type on measurements.

In this study, the disc margins, base plane, and reference plane were set based on the BMO. Previously, calculating the distance between the BMO and ILM (i.e., the BMO-MRW) was found to be more useful for diagnosing glaucoma and determining its progression than identifying the rim with the naked eye. [19] Taken together, these results show that evaluating the ONH based on BMO-set parameters is a promising technique, because it reflects the unique characteristics of each optic disc type. Thus, the BMO can be used as an orientation point in the objective classification of the ONH.

We found that the SS-OCT-based parameters of disc topography described in this study had excellent reproducibility. Moreover, examinations based on SD-OCT have also shown excellent reproducibility, with a CV ranging from 1.1 to 7.6% for intra-visit measurements and from 1.1% to 11.7% for inter-visit measurements. The cup/disc area ratio had the lowest CV (1.1%) in both types of measurement. [29] Thus, the evaluation of cupping parameters with OCT has shown excellent reproducibility and can be considered a suitable part of glaucoma assessment.

Glaucoma causes irreversible loss of vision, making early diagnosis and evaluation of the ONH very important. Accurate examination data, such as that provided by SS-OCT, are a key part of early diagnosis. Furthermore, reliable methods for disc classification are also important, because of differing clinical characteristics in patients with each disc type (i.e., Nicolela’s 4 types) and the impact on the pattern of progress. [22, 30] However, classification of the ONH according to Nicolela’s methods is affected by variations arising from the subjectivity of judgments made based on funduscopic observations. [21] Thus, an objective, highly reproducible method for disc classification is an important goal for improving individual treatment of glaucoma. In this variation study, our objective method enabled us to determine disc type with an 80.0% identification rate overall. Furthermore, the identification rate was 81.1% in a subset of the validation group that contained 24 early glaucoma eyes, showing that our prediction formula was valid for disc type classification in early-stage glaucoma. Early diagnosis and initiation of glaucoma treatment are critical for maintaining a good quality of life in glaucoma patients. Thus, our accurate, reproducible method of disc classification should improve long-term glaucoma care and influence the selection of treatment strategies for glaucoma.

Recently, we investigated the objective classification of ONH type based on stereo photography in the Glaucoma Stereo Analysis Study, a multi-institutional joint glaucoma research group. [5] This study used stereo fundoscopy, a method that enabled objective measurement of the ONH in images similar to those used in normal fundoscopy. Thus, clinical findings from objective OCT-based disc type classification promise to complement findings obtained from clinical background and pathology, such as high IOP, elongated axial length, spasm, and ischemia.

This study had some limitations. It was cross-sectional and included subjects of only a single ethnicity (Japanese). Additionally, it is difficult to use OCT data to quantify undermining of the cup and or the morphology of regions located beneath the central retinal vessels, because of attenuation in the OCT signal. Highly myopic eyes with large peripapillary atrophy cause further difficulties, because the BMO cannot be clearly detected in these eyes. In approximately 10% of eyes, disc parameters cannot be calculated because of complicated disc morphology. Thus, although the morphological variety of the optic disc in individuals was a disadvantage in this study, disc type remains a critical location for the diagnosis of glaucoma. Furthermore, the disadvantage is minimal in clinical practice, because ONH morphological data are considered together with data on RNFL defects and other internal layer defects, in both peripapillary and macular lesions.

## Conclusions

In conclusion, we developed a new, highly reproducible, objective method to classify the disc type of eyes with glaucoma, based on SS-OCT measurements. We found that 120 μm was the optimal reference plane height in this analysis to minimize the effect of optic disc type. Moreover, we found that using these parameters to classify the optic disc type of glaucomatous eyes provided results that coincided well with those of previous, subjective classification methods. Thus, objective disc type classification based on SS-OCT data may open new avenues, not only for future, more in-depth research on glaucoma, but also in clinical care for glaucoma.

## Supporting Information

S1 Table The subject list for reference plane analysis. (XLSX)

S2 Table The subject list for validation analysis. (XLSX)

S3 Table The subject list for reproducibility analysis. (XLSX)
